# Multiple major increases and decreases in mitochondrial substitution rates in the plant family Geraniaceae

**DOI:** 10.1186/1471-2148-5-73

**Published:** 2005-12-20

**Authors:** Christopher L Parkinson, Jeffrey P Mower, Yin-Long Qiu, Andrew J Shirk, Keming Song, Nelson D Young, Claude W dePamphilis, Jeffrey D Palmer

**Affiliations:** 1 Department of Biology, Indiana University, Bloomington, IN 47405-3700, USA; 2 Department of Biology, University of Central Florida, Orlando, FL 32816, USA; 3 Department of Ecology and Evolutionary Biology, University of Michigan, Ann Arbor, MI 48109, USA; 4 Department of Otolaryngology, University of Washington, Seattle, WA, 98195, USA; 5 Sigma Chemical Co., 3300 S. 2nd St., St. Louis, MO, 63118, USA; 6 Department of Biology, Holyoke Community College, Holyoke, MA 01040, USA; 7 Department of Biology, Penn State University, University Park, PA 16802-0001, USA

## Abstract

**Background:**

Rates of synonymous nucleotide substitutions are, in general, exceptionally low in plant mitochondrial genomes, several times lower than in chloroplast genomes, 10–20 times lower than in plant nuclear genomes, and 50–100 times lower than in many animal mitochondrial genomes. Several cases of moderate variation in mitochondrial substitution rates have been reported in plants, but these mostly involve correlated changes in chloroplast and/or nuclear substitution rates and are therefore thought to reflect whole-organism forces rather than ones impinging directly on the mitochondrial mutation rate. Only a single case of extensive, mitochondrial-specific rate changes has been described, in the angiosperm genus *Plantago*.

**Results:**

We explored a second potential case of highly accelerated mitochondrial sequence evolution in plants. This case was first suggested by relatively poor hybridization of mitochondrial gene probes to DNA of *Pelargonium hortorum *(the common geranium). We found that all eight mitochondrial genes sequenced from *P. hortorum *are exceptionally divergent, whereas chloroplast and nuclear divergence is unexceptional in *P. hortorum*. Two mitochondrial genes were sequenced from a broad range of taxa of variable relatedness to *P. hortorum*, and absolute rates of mitochondrial synonymous substitutions were calculated on each branch of a phylogenetic tree of these taxa. We infer one major, ~10-fold increase in the mitochondrial synonymous substitution rate at the base of the *Pelargonium *family Geraniaceae, and a subsequent ~10-fold rate increase early in the evolution of *Pelargonium*. We also infer several moderate to major rate decreases following these initial rate increases, such that the mitochondrial substitution rate has returned to normally low levels in many members of the Geraniaceae. Finally, we find unusually little RNA editing of Geraniaceae mitochondrial genes, suggesting high levels of retroprocessing in their history.

**Conclusion:**

The existence of major, mitochondrial-specific changes in rates of synonymous substitutions in the Geraniaceae implies major and reversible underlying changes in the mitochondrial mutation rate in this family. Together with the recent report of a similar pattern of rate heterogeneity in *Plantago*, these findings indicate that the mitochondrial mutation rate is a more plastic character in plants than previously realized. Many molecular factors could be responsible for these dramatic changes in the mitochondrial mutation rate, including nuclear gene mutations affecting the fidelity and efficacy of mitochondrial DNA replication and/or repair and – consistent with the lack of RNA editing – exceptionally high levels of "mutagenic" retroprocessing. That the mitochondrial mutation rate has returned to normally low levels in many Geraniaceae raises the possibility that, akin to the ephemerality of mutator strains in bacteria, selection favors a low mutation rate in plant mitochondria.

## Background

For almost 20 years, it has been widely appreciated that nucleotide substitution rates are unusually low in mitochondrial genomes of land plants. This conclusion was first reached by Wolfe et al. [1] from an examination of synonymous substitution rates for several mitochondrial genes among a small set of angiosperms. Wolfe et al. [1] estimated that synonymous substitution rates in plant mitochondrial genomes are several times lower than in chloroplast genomes, 10–20 times lower than in plant nuclear genomes, and 50–100 times lower than in mammalian mitochondrial genomes. Palmer and Herbon [2] quickly extended this inference to the entire mitochondrial genome (most of which is noncoding) through genome-wide comparative restriction site mapping among crucifers and also showed that whereas sequence evolution is abnormally slow in plant mitochondrial genome, structural evolution is quite rapid.

Subsequent studies have extended this picture of unusually low mitochondrial substitution rates to other groups of land plants [reviewed in 3–5]. Within this context of generally slow evolution, a few studies have reported moderate variation in mitochondrial synonymous substitution rates between different groups of plants [6-9]. In most of these cases, correlated rate changes have been noted for chloroplast and/or nuclear genes. For example, all three genomes of grasses exhibit several-fold higher synonymous rates than do palms [6]. These findings suggest the operation of forces, such as generation time effects [6-8], paternal transmission of organelles [9], or correlated substitution and speciation rates [10,11], acting on all three genomes or on both organellar genomes.

Beginning in the late 1990's, we have carried out a large-scale Southern hybridization survey in which numerous mitochondrial and chloroplast gene and intron probes were hybridized to filter blots containing total DNAs from 280 diverse angiosperms [12-16]. With the exception of two plants, most if not all of the mitochondrial gene probes hybridized well to all 280 plant DNAs. For these two plants, all mitochondrial probes hybridized poorly if at all, despite typically strong hybridization with chloroplast probes. This very reduced mitochondrial hybridization (relative to chloroplast hybridization) suggested either 1) greatly reduced mitochondrial genome copy number in one or both plants, 2) highly elevated mitochondrial sequence divergence, or 3) loss from the mitochondrial genome and transfer of these genes to the low copy number and high mutation rate environment of the nucleus. A preliminary report [15] provided evidence of exceptional divergence for two, normally mitochondrially-located genes for each of these two plants, *Plantago rugelii *(plantain) and *Pelargonium hortorum *(the common geranium).

In a recent study [17], we explored the *Plantago *case in some detail, showing that it represents the first example of extremely rapid sequence evolution of mitochondrial DNA in plants. This rapid evolution was confined to the mitochondrial genome and to *Plantago*, for which major increases and decreases were found in the mitochondrial synonymous rate and, underlying this, the mitochondrial mutation rate. The fastest evolution in *Plantago *was estimated to exceed even the fastest evolution in animal mitochondria by an order of magnitude. Coupled with the discovery of other angiosperms with exceptionally slow evolution (even for plants), synonymous substitution rates in angiosperm mitochondrial genomes were estimated to vary by a factor of some 4,000-fold [17].

Here we explore the *Pelargonium *situation and report a second, entirely independent case of exceptionally rapid and variable sequence evolution in plant mitochondria, involving the Geraniaceae and especially *Pelargonium*. The Geraniaceae and *Plantago *cases are similar in overall aspect, involving initial, stepwise major increases in the rate of mitochondrial synonymous substitutions, followed by major rate decreases in certain descendant lineages. There are, however, differences between the two cases, including striking evidence from the Geraniaceae that high mitochondrial mutation rates may be deleterious in plants, at least in the long run.

## Results

We used PCR to isolate nearly full-length segments of seven mitochondrial genes from *Pelargonium hortorum *and three genes from *P. reniforme*. All seven *P. hortorum *mitochondrial genes show exceptional divergence relative to other angiosperms. This finding suggests that exceptionally rapid mitochondrial evolution – rather than reduced mitochondrial DNA copy number or mitochondrial gene loss/nuclear transfer – is the explanation for the very poor hybridization of heterologous mitochondrial gene probes to *P. hortorum *total DNA in the survey blots described in Background. Several other lines of evidence support the conclusion that these genes are located in the mitochondrial genome and are functional: 1) Two of the seven genes were tested by Southern blots and shown to hybridize preferentially to purified mitochondrial DNA compared to total DNA from *P. hortorum *(data not shown). 2) Both genes tested by Northern blots hybridized preferentially to poly (A)- RNA compared to poly (A)+ RNA (data not shown), and all three genes examined for RNA editing are transcribed. 3) Four of the seven genes are invariantly mitochondrially-located in all examined eukaryotes (except in those rare organisms where the gene function has been dispensed with entirely). 4) The five protein genes examined all contain intact open reading frames. 5) In comparisons with homologs from other Geraniaceae (see below), the protein genes all display very low ratios of nonsynonymous to synonymous substitutions, indicating that they are functional and evolving under strong purifying selection.

**Figure 1:**
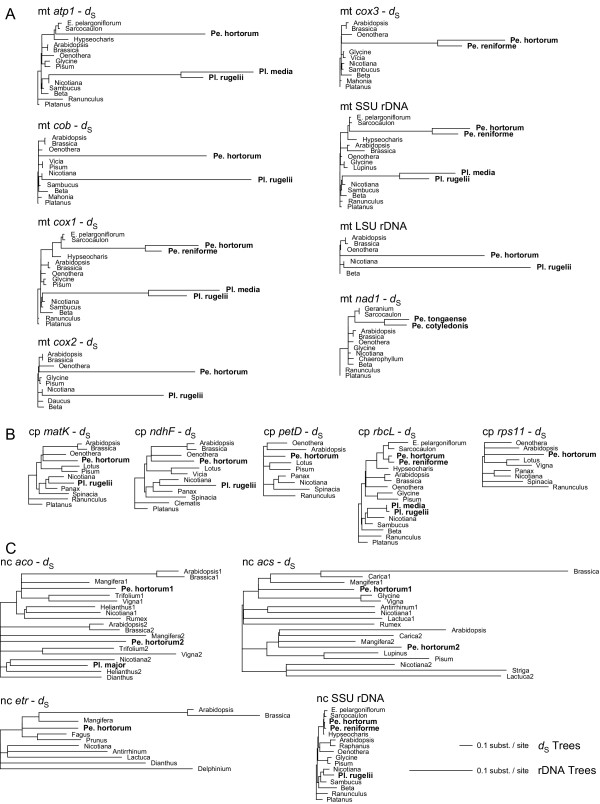
**Extreme divergence of mitochondrial genes in *Pelargonium hortorum***. Shown are ML trees based on synonymous (*d*_S_) sites for protein genes or all sites for rRNA genes. All tree topologies were completely constrained as described in Methods. All *d*_S _trees are drawn to one scale, while all rDNA trees are drawn to a different scale (see bottom right). Abbreviations: Pe, *Pelargonium*; Pl, *Plantago*; E, *Erodium*. (**A**) Mitochondrial gene trees are based on 1,275 (*atp1*), 1,119 (*cob*), 1,413 (*cox1*), 723 (*cox2*), 690 (*cox3*), 805 (LSU rDNA), 1,395 (SSU rDNA), and 198 (*nad1*) NT. (**B**) Chloroplast gene trees are based on 1,497 (*matK*), 2,172 (*ndhF*), 483 (*petD*), 1,377 (*rbcL*), and 417 (*rps11*) NT. (**C**) Nuclear gene trees are based on 945 (aco, 1-aminocyclopropane-1-carboxylate oxidase), 1,245 (acs, 1-aminocyclopropane-1-carboxylate synthase), 2,226 (etr, ethylene receptor), and 1,667 (SSU rDNA) NT.

Because a disproportionate amount of the enhanced divergence of the protein genes was at synonymous sites, we chose to present their divergence graphically in the form of phylogenetic trees constructed from synonymous sites only (Fig. 1). The level of divergence in *P. hortorum *is generally comparable, at both synonymous sites and all sites for ribosomal RNA genes, to divergences of *Plantago rugelii *(Fig. 1), which has one of the most divergent mitochondrial genomes in *Plantago *[17] (*P. media *is the most divergent *Plantago*). For the three genes available, *Pelargonium reniforme *is also quite divergent but consistently less so than *P. hortorum *(Fig. 1). For the eighth mitochondrial gene shown in Fig. 1, *nad1*, only a relatively short sequence was available, and this only from different species of *Pelargonium *[18]. To illustrate synonymous site divergence for this gene, we chose *P. tongaense *and *P. cotyledonis*, as these are close relatives of *P. hortorum *and *P. reniforme*, respectively. The less exceptional apparent divergence of *nad1 *for these two taxa (Fig. 1) is probably a function of the very short region analyzed (198 NT in total, and only about 50 synonymous sites) rather than gene-specific differences in substitution rates. Phylogenetic analysis of synonymous sites for five chloroplast and three nuclear genes shows, unlike the mitochondrial situation, no evidence of exceptional divergence in *P. hortorum *(Fig. 1).

**Figure 2:**
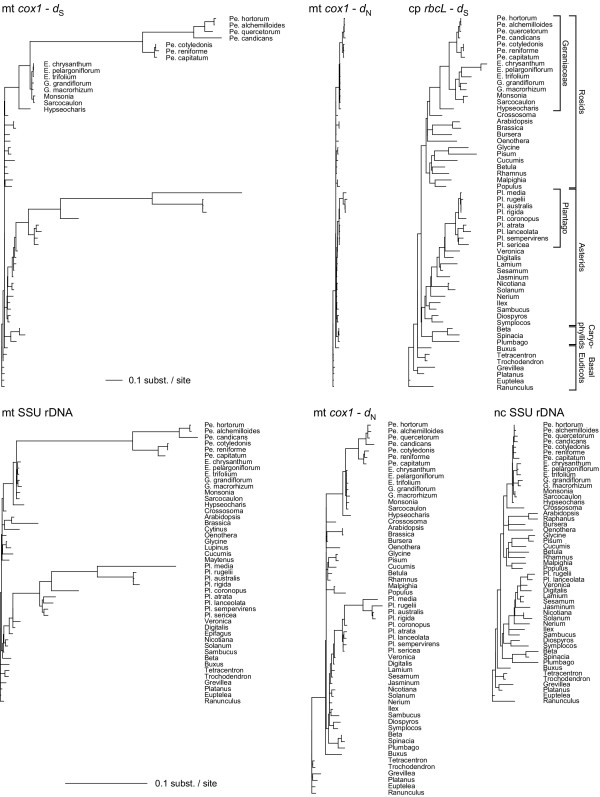
**Extensive rate variation in Geraniaceae mitochondrial genes**. Shown are ML trees based on synonymous (*d*_S_) or nonsynonymous (*d*_N_) sites for protein genes or all sites for rRNA genes. All tree topologies were completely constrained as described in Methods. To emphasize branch length disparities, taxon names were moved to the right of each tree. Unlabeled branches in the *cox1 *trees are the same species shown for *rbcL*. The top three trees are drawn to the same scale, while the bottom three trees are drawn at five times that scale (see scale bars). Abbreviations: Pe, *Pelargonium*; Pl, *Plantago*; E, *Erodium*; G, *Geranium*. Gene trees are based on 1,413 (cox1), 1,377 (rbcL), 1,471 (mitochondrial SSU rDNA), and 1,667 (nuclear SSU rDNA) NT.

An expanded data set (Fig. 2) was generated for two mitochondrial genes, *cox1 *and small subunit ribosomal DNA (SSU rDNA), in order to explore the extent and pattern of mitochondrial rate heterogeneity within *Pelargonium *and other genera in the Geraniaceae, and to allow comparison with rate variation in other rosids and across eudicots, including the previously described case of rapid mitochondrial evolution in *Plantago *[17]. Seven species of *Pelargonium *were examined, as were eight other Geraniaceae species representing the five other genera in the family.

The fifteen Geraniaceae taxa all show enhanced divergence at *cox1 *synonymous sites compared to all other rosids examined, but to markedly different extents, falling into three divergence groups (Fig. 2). The five non-*Pelargonium *genera are divergent compared to other rosids, members of *Pelargonium *subgenus *Pelargonium *(comprising *P. cotyledonis *through *P. capitatum*) are much more divergent, and those of the other subgenus, *Ciconium *(comprising *P. hortorum *through *P. candicans*), are even more divergent. The mitochondrial SSU rDNA gene shows much the same pattern, which is striking considering that all sites are included for this gene compared to only synonymous sites for *cox1*. The magnitude and overall aspect of enhanced mitochondrial sequence divergence in Geraniaceae – different levels of divergence according to taxonomic group within the family – mirrors the pattern seen within *Plantago *(Fig. 2) [17]. Consistent with the results shown in Fig. 1 for *Pelargonium hortorum*, levels of chloroplast synonymous site divergence and nuclear SSU rDNA divergence are unexceptional in Geraniaceae (except for somewhat enhanced *rbcL *divergence in *Erodium*).

**Figure 3:**
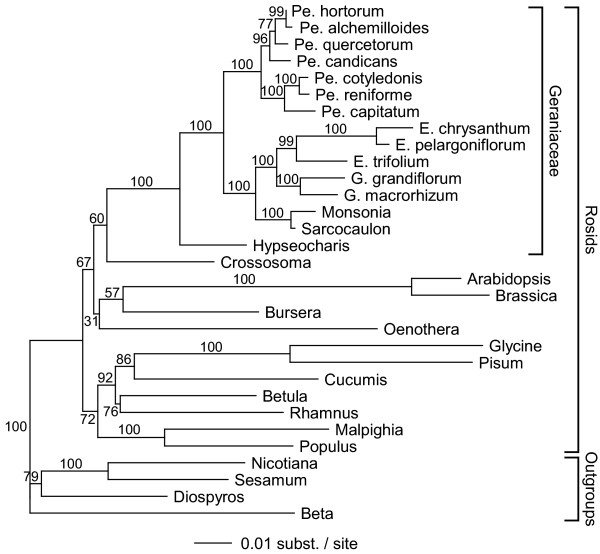
**Multigene phylogeny of Geraniaceae and other rosids**. Shown is a ML tree based on a 6,226 NT alignment comprising three chloroplast genes (*atpB*, *matK*, *rbcL*) and the nuclear SSU rDNA. Numbers on branches are ML bootstrap values. The tree is rooted using one caryophyllid (*Beta*) and three asterids. Abbreviations: Pe, *Pelargonium*; E, *Erodium*; G, *Geranium*.

To put the synonymous site divergence data on a quantitative footing, we calculated absolute rates of *cox1 *synonymous substitutions along all branches in the rosid part of the *cox1 *trees shown in Fig. 2. Absolute rates were calculated for each branch by dividing its synonymous branch length by the estimated divergence time of the branch. Divergence times within rosids were calculated using the 4-gene (three chloroplast and one nuclear), 6,226 NT character set used to estimate rosid phylogeny in Fig. 3. Support for the relationships in this phylogenetic tree is mixed at basal nodes within rosids. Critically, though, support is strong at the node leading to Geraniaceae and throughout the family (96–100% bootstrap support at 13 of 14 nodes; Fig. 3).

**Figure 4:**
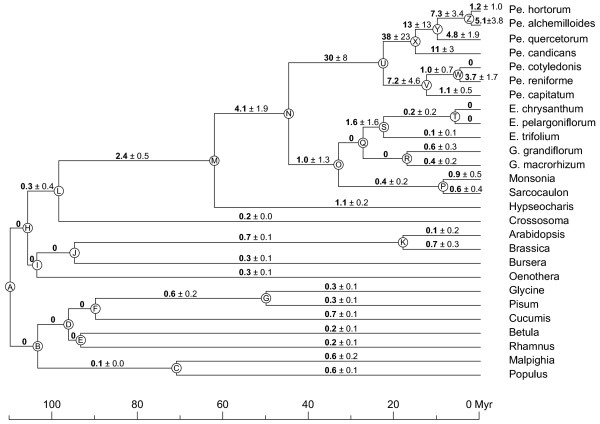
**Variable rates of synonymous substitutions in Geraniaceae *cox1 *genes**. Shown is a chronogram based on the topology of Fig. 3. Nodes are labeled A-Z (also see Additional File 1). Shown above each branch are *cox1 R*_S _values (*R*_S _= absolute rate of synonymous substitution per branch) in SSB (sub/site/byr). Abbreviations: Pe, *Pelargonium*; E, *Erodium*; G, *Geranium*. See Additional File 1 for the full set of *R*_S _values taken to two digits after the decimal point, plus full sets of *R*_N_, *d*_S_,*d*_N_,*R*_N_/*R*_S_, and divergence time values.

A chronogram (time-based tree) of rosids is shown in Fig. 4, with *R*_S_, the absolute rate of *cox1 *synonymous substitutions, marked on each branch in units of substitutions per site per billion years (SSB units). The dearth of inferred synonymous site changes on the relatively short branches at the base of rosids is reflected in the *R*_S _= 0 estimates on most of these branches. *R*_S _is comparably low (0.31 SSB) on the H-L branch leading to the common ancestor of Geraniaceae and *Crossosoma *as throughout the rest of the non-zero rosid branches (*R*_S _= 0.14–0.74 SSB). On the next four branches leading from the H-L branch towards the "top" of tree, *R*_S _becomes progressively higher, from 0.3→2.4→4.1→30→38 SSB, before diminishing on subsequent branches. There is no reason to think that rate changes should coincide in timing with cladogenetic events. Given this, and wishing to minimize the number of inferred rate changes, we can very provisionally model these *branch-wise *rate increases with a minimum of two actual rate changes: an ~10-fold increase in *R*_S _(from 0.3 to 4 SSB) in the common ancestor of the Geraniaceae (L-M branch) and a further ~10-fold increase (from 4 to 38) in the common ancestor of *Pelargonium *(N-U; Fig. 4). Thus, we provisionally infer at least (see Discussion) a 100-fold overall increase in *R*_S _from near the base of rosids to the period of putatively fastest evolution in *Pelargonium.*

The terminal branches leading to all examined species of *Pelargonium *have estimated *R*_S _values considerably below the genus' peak inferred value of 38 SSB. Likewise, the terminals leading to all non-*Pelargonium *Geraniaceae have *R*_S _values considerably lower than 4 SSB. In fact all of this latter set of terminals have values comparable to the inferred ancestral rosid value of roughly 0.3 SSB, as do even some of the *Pelargonium *terminals (Fig. 4). Thus, there must have been multiple decreases in the synonymous substitution rate, some of considerable magnitude, following its early increases. We are confident of at least three rate decreases in the Geraniaceae: one each in the two subgenera of *Pelargonium *and one in the common ancestor of the clade comprising *Erodium *through *Sarcocaulon*. Furthermore, Fig. 4 raises the possibility of additional rate decreases, e.g, in *P. hortorum*, in *Erodium*, and in *Hypseocharis*.

The large standard errors on many of the *R*_S _values within the family (Fig. 4) pose a particular problem in formulating rate-change scenarios with any certainty. But what is clear is that there have been multiple increases and decreases in the rate of mitochondrial synonymous substitutions within the Geraniaceae, and that the overall magnitude of these changes has been quite large.

Divergence at nonsynonymous sites in *cox1 *was also analyzed phylogenetically and is plotted both at the same scale as synonymous site divergence (Fig 2, middle top) and at a five-fold expanded scale (Fig. 2, middle bottom). Although replacement sites also show enhanced divergence in *Pelargonium *and other Geraniaceae relative to other rosids, the effect is considerably muted compared to the pattern of synonymous site divergence. Absolute rates of nonsynonymous substitutions (*R*_N_) were calculated within the rosids and are given in Additional File 1, as are *R*_N_/*R*_S _values. Among the very lowest *R*_N_/*R*_S _values are those on the branches with the highest *R*_S _values (*R*_N_/*R*_S _= 0.02 for the N-U branch of *R*_S _= 30, and *R*_N_/*R*_S _= 0.03 for the U-X branch of *R*_S _= 38).

One notable feature of the very rapidly evolving mitochondrial DNAs of mammals is a pronounced (ca. 20-fold) transition/transversion (ti/tv) bias in favor of transitions [19]. The most divergent Geraniaceae *cox1 *genes, in *Pelargonium*, show only a modest increase in ti/tv (= 1.91 averaged across all within-*Pelargonium *comparisons) relative to all non-Geraniaceae (ti/tv = 0.82). Base composition and the overall mutational spectrum are not significantly different in *Pelargonium *than in other plant lineages (data not shown).

Although only limited data are available, there seems to be relatively little RNA editing of mitochondrial genes in the Geraniaceae (Table 1). We directly assessed RNA editing, by cDNA sequencing, for three mitochondrial genes in *P. hortorum*. Only a single, C→U edit site was found among the three genes, and very few edits were predicted (Table 1) for the various other sequenced Geraniaceae mitochondrial genes using a sensitive program [20] for predicting C→U editing of angiosperm mitochondrial genes. In comparison, other rosids (*Crossoma *through *Pisum *in Table 1), as well as the caryophyllid *Beta *and the grasses *Oryza *and *Triticum*, generally have much more RNA editing of these same five protein genes, with many edit sites conserved among angiosperms.

**Table 1:** Observed and predicted number of RNA editing sites

**Organism**	***atp1***	***cob***	***cox1***	***cox2***	***cox3***	**Editing Site Source**
*Pe. hortorum*	1	**1**	**0**	**0**	1	this study
*Pe. alchemilloides*	-	-	1	-	-	
*Pe. quercetorum*	-	-	0	-	-	
*Pe. candicans*	-	-	0	-	-	
*Pe. cotyledonis*	-	-	0	-	-	
*Pe. reniforme*	-	-	0	-	2	
*Pe. capitatum*	-	-	0	-	-	
*E. pelargoniflorum*	0	-	0	-	-	
*G. macrorhizum*	-	-	0	-	-	
*Sarcocaulon*	0	-	0	-	-	
*Hypseocharis*	0	-	1	-	-	
*Crossosoma*	-	-	22	-	-	
*Arabidopsis*	**5**	**7**	**0**	**15**	**8**	GenBank: Y08501, Y08502
*Brassica*	**5**	**8**	**1**	**13**	**7**	GenBank: AP006444
*Oenothera*	**4**	**14**	17	**15**	12	GenBank: X04023, X07126, X00212
*Glycine*	1	-	17	13	11	
*Pisum*	1	15	18	**13**	-	GenBank: X52866
*Beta*	**3**	**13**	**0**	**9**	**4**	JPM and JDP, unpublished
*Oryza*	**5**	**19**	**4**	**19**	**1**	GenBank: BA000029
*Triticum*	**6**	**18**	4	**14**	**13**	GenBank: X80470, X02352, X52867, X52539

Numbers in bold indicate that editing sites were identified experimentally. All other reported numbers were predicted with PREP-Mt [20] using a cutoff value of 0.5.

## Discussion

### Tempo and pattern of rate variation

We have discovered dramatic variation in rates of mitochondrial synonymous substitutions within the angiosperm family Geraniaceae. Our principal findings are three-fold: 1) The synonymous rate increased hugely during the evolution of the Geraniaceae, by at least a factor of 100. 2) This overall rate increase occurred via multiple (at least two, possibly more) stepwise and temporally separated rate increases. 3) Following these rate increases, at least three, and quite possibly more, rate decreases occurred, with many lineages reverting to the very low synonymous rates typical of most angiosperms.

Our estimates for the highest and lowest rates of synonymous substitutions, and of the number of rate changes, are inherently conservative ones, being constrained by the limited taxonomic sampling of this study. The *R*_S _values by definition average the substitution rate across an entire branch, the length/time of that branch being a function of the number of extant Geraniaceae taxa and our sampling of those taxa. Any one *R*_S _value may in fact average across periods of substantially higher and lower absolute substitution rate than would be indicated by the *R*_S _value itself. For example, the highest branch-wise (*R*_S_) rate estimated in this study, of 38 SSB, may turn out to be a significant underestimate of the fastest period of mitochondrial evolution within *Pelargonium*. We sampled only 7 of ~280 species in the genus, and thus there is ample opportunity to more finely dissect the pattern of rate variation and thereby discover greater peaks (and valleys) of substitution rates, as well as detect a larger number of rate changes.

If synonymous rates are more or less equal across genes within a mitochondrial genome (as seems to be the case for *P. hortorum*; Fig. 1), then sampling multiple protein genes (and among many taxa) should provide another opportunity to better elucidate the tempo and pattern of rate changes. In particular, this should significantly reduce the standard errors on the estimates of *R*_S_, many of which are quite high for *cox1 *alone (Fig. 4 and Additional File 1). Sequencing additional non-mitochondrial genes, to reduce the standard errors on the divergence time estimates (see Additional File 1), should also be helpful in this regard.

The mitochondrial rate variation in Geraniaceae uncovered in this study is similar in several respects to that recently described in *Plantago *[17]. In both cases, 1) there is dramatic variation in synonymous substitution rates; 2) taxa fall into multiple (three or four) groups with respect to the amount of accumulated sequence divergence (Fig. 2); 3) the most divergent groups have accumulated comparable levels of synonymous site divergence (Fig. 2); 4) at least two successive major increases in the synonymous rate occurred relatively early on; 5) multiple rate decreases occurred subsequent to the initial increases; and 6) rates of chloroplast and nuclear evolution are not elevated, such that in the fastest lineages mitochondrial rates significantly exceed those of the chloroplast and nuclear genomes (Fig. 2; ref. [17]).

The Geraniaceae and *Plantago *situations also differ in several ways: 1) The Geraniaceae situation encompasses a broader group taxonomically. 2) The initial speed-up occurred much earlier in the Geraniaceae (ca. 80 Myr) than in *Plantago *(ca. 30 Myr). 3) The peak rates estimated in *Plantago *(ca. 200 SSB and possibly even 700 SSB) far exceed those in Geraniaceae (38 SSB), although this difference could in large part be a sampling artifact (i.e., if the fastest period of mitochondrial evolution extended throughout a single sampled branch in *Plantago*, whereas that in *Pelargonium *was averaged, on the fastest measured branch, with periods of significantly slower evolution). 4) There seems to be less of a tendancy to revert to a typically low substitution rate (of ca. 0.5 SSB) in *Plantago *compared to Geraniaceae. Overall, then, the Plantago speed-up is more recent, apparently more intense, and apparently less reverted. Like *Pelargonium*, *Plantago *is a large genus (in fact the two are almost exactly the same size), and it will be interesting to see how the two situations compare once more taxa and genes (and even some complete genomes) are sampled from both groups.

Geraniaceae and *Plantago *are, to our knowledge, the only two cases of such dramatic variation in synonymous substitution rates – both in magnitude and rollercoaster-like behavior – documented in any group of plant mitochondrial genomes, or, for that matter, in any genome/group whatsoever. However, several published, mostly phylogenetic studies have identified other plants for which mitochondrial divergence is disproportionately high compared to chloroplast divergence. These long-branched mitochondrial clades include *Ephedra *in the Gnetales [21-23], the monocots *Acorus *and Alismatales [24], the lycophyte *Selaginella *[25], the asterids *Goodenia *and *Polemonium *[17,26], and the simple thalloid and leafy liverworts [27]. With the exception of Goodenia [17], none of these cases has been quantitatively treated, and the published trees include all sites rather than just synonymous sites. Nonetheless, in each case, the accumulated divergence appears to be lower than in *Pelargonium *or *Plantago*. However, in most of these cases, only one or two species were examined, and thus there is plenty of opportunity to find even more divergent lineages in these groups that may approach or even exceed *Pelargonium *and *Plantago *in amount of divergence and peak substitution rate.

### Mutation rates, mechanisms, and selection

Because the extensive variation in substitution rates in the Geraniaceae is restricted to the mitochondrial genome and because nonsynonymous rates are proportionately less elevated than synonymous rates (Fig. 2, Additional File 1), the root cause of this rate variation is almost certainly changes in the mitochondrial mutation rate. The same conclusion was reached for *Plantago *mitochondrial rate variation [17], whereas most other cases of (much more modest) synonymous site variation in plant mitochondrial DNA have been ascribed to factors that would affect all three plant genomes, such as generation time, population size, and/or speciation rate [6-8].

By analogy to the extensive literature on mutators (and antimutators) in bacteria and yeast, mutations affecting a broad range of processes that impinge on the mutation rate could be responsible for the extensive variation in mitochondrial mutation rates in Geraniaceae. The only mitochondrial mutators characterized thus far, in lab mutants of the yeast *Saccharomyces cerevisiae*, affect DNA replication and mismatch repair [28]. These are also the most common mutators in microorganisms [29,30]. In plants and virtually all other mitochondrial systems, these processes are controlled entirely by nuclear genes. Thus, the major increases in the mitochondrial mutation rate that took place early in the evolution of the Geraniaceae and then *Pelargonium *could be the consequence of nuclear mutations resulting in error-prone replication or defective mismatch repair, as postulated for other cases of synonymous rate variation in plant organelle genomes [7,17,31]. Subsequent rate decreases could reflect direct reversal or compensatory suppression of these mutator mutations. Mitochondrial-specific forces could also be at work, e.g., major increases in the amount of oxygen free-radical damage to mitochondrial DNA [32,33].

That Geranicaeae mitochondrial genes seem, based on limited samping (Table 1), to be relatively bereft of RNA editing, raises the possibility of a novel molecular mechanism, which we term "mutagenic retroprocessing" (see also [34]). Reverse transcriptases are extremely error-prone polymerases, and so exceptionally high levels of reverse transcription in Geraniaceae mitochondria, coupled with high levels of homologous recombination, could in principle be responsible for at least part of the elevation in mitochondrial mutation rate seen in the family. If this hypothesis is correct, we would expect nontranscribed regions to be much less divergent than genes. Unfortunately, identifying and amplifying homologous intergenic spacer DNA is not straightforward given the high rate of rearrangement in plant mitochondrial genomes [2], and our attempts to do so in *Pelargonium *have been unsuccessful. According to this hypothesis, the existence of limited RNA editing in extant Geraniaceae (Table 1) would reflect mutational drift to unedited C residues following the evident reversals in mutation rate, i.e, cessation of the hypothetical mutagenic retroprocessing activity.

In all 15 examined Geraniaceae, the mitochondrial mutation rate has dropped from its peak levels, with at least three (and probably more) separate rate decreases implied by the *R*_S _patterns of Fig. 4. In many taxa, the mutation rate appears to have dropped more or less to the low rates (ca. 0.3 SSB) typical of most rosid lineages (Fig. 4). And even those taxa (four species of *Pelargonium*) whose terminal branches possess relatively high *R*_S _values may nonetheless have more fully reverted mutation rates, the present-day rates being averaged with potentially higher rates earlier on these branches.

Such a pervasive local pattern of rate decreases, combined with pervasively slow mitochondrial evolution across land plants in general, suggests that high mutation rates are selected against in the evolution of plant mitochondrial genomes. Chance mutations that directly reverse or otherwise suppress the earlier mutator-like mutations in Geraniaceae may be favored and fixed by natural selection. From this perspective, plant mitochondrial mutators may be viewed as ephemeral, like bacterial mutators but on a much longer time scale.

If most or all Geraniacease have reverted to low mitochondrial mutation rates, then it may be difficult if not impossible to figure out the nature of the mutator mutations that caused the rapid periods of mitochondrial evolution. Mutagenic retroprocessing, admittedly a long-shot hypothesis, is nonetheless attractive in this regard because it would leave obvious signatures in the genome (see above).

## Methods

### Molecular techniques and sources of DNA sequences

Methods for plant DNA isolation, Southern and Northern blot hybridization, PCR isolation, DNA cloning, cDNA preparation, and DNA sequencing are as in [12,13,35]. Additional sequences used in this study were taken from GenBank and are listed in Additional File 2, as are GenBank numbers for the sequences generated in this study. PCR primer sequences and aligned data sets are available upon request.

### Phylogenetic analysis

Phylogenetic relationships within the rosids were determined from a concatenated data set consisting of nuclear SSU rDNA and the chloroplast genes *atpB*, *matK*, and *rbcL*. Total aligned length was 6,226 NT. Poorly alignable regions and regions with gaps in most taxa were excluded from the analyses. A maximum likelihood (ML) tree was constructed with PAUP*, version 4.0b10 [36], by using the general time-reversible model, a gamma distribution with four rate categories, and an estimate of the proportion of invariant sites. The rate matrix, base frequencies, shape of the gamma distribution, and proportion of invariant sites were estimated before the ML analysis from a parsimony tree constructed from the data. Support for the ML tree was evaluated by the bootstrap procedure with 500 replicates using parameters estimated from the ML tree.

### Divergence time estimates

Divergence times for all nodes within rosids were calculated using a penalized likelihood approach as implemented in the r8s program [37]. A fixed time constraint of 110 million years [38,39] was used for the crown group age of rosids. The ML tree from the phylogenetic analysis of rosids (Fig. 3) was used as the starting tree. A smoothing factor of 18 was determined by using the r8s cross-validation procedure [37]. Different starting points of initial age estimates and reanalysis after perturbation of the final age estimates had no effect on the results. Standard errors for the divergence time of each node were calculated by rerunning the divergence time analyses on 500 bootstrapped data sets.

The elapsed time along each branch, *T*_*ij*_, was calculated as the difference in divergence times of the starting node, *T*_*i*_, and the ending node, *T*_*j*_, that define the branch. Standard errors for *T*_*ij *_were calculated according to the formula

σTij=(σTi)2+(σTj)2
 MathType@MTEF@5@5@+=feaafiart1ev1aaatCvAUfKttLearuWrP9MDH5MBPbIqV92AaeXatLxBI9gBaebbnrfifHhDYfgasaacH8akY=wiFfYdH8Gipec8Eeeu0xXdbba9frFj0=OqFfea0dXdd9vqai=hGuQ8kuc9pgc9s8qqaq=dirpe0xb9q8qiLsFr0=vr0=vr0dc8meaabaqaciGacaGaaeqabaqabeGadaaakeaacqaHdpWCdaWgaaWcbaGaemivaq1aaSbaaWqaaiabdMgaPjabdQgaQbqabaaaleqaaOGaeyypa0ZaaOaaaeaacqGGOaakcqaHdpWCdaWgaaWcbaGaemivaq1aaSbaaWqaaiabdMgaPbqabaaaleqaaOGaeiykaKYaaWbaaSqabeaacqaIYaGmaaGccqGHRaWkcqGGOaakcqaHdpWCdaWgaaWcbaGaemivaq1aaSbaaWqaaiabdQgaQbqabaaaleqaaOGaeiykaKYaaWbaaSqabeaacqaIYaGmaaaabeaaaaa@43E8@

### Branch length estimates

Branch lengths, representing the number of substitutions per synonymous site (*d*_S_) or number of substitutions per nonsynonymous site (*d*_N_), were determined for protein genes using codeml in the PAML package, version 3.14 [40]. The Muse-Gaut (MG94) codon model was used with separate *d*_N_/*d*_S _ratios for each branch. Codon frequencies were computed by using the F3 × 4 method. The transition/transversion rate ratio and *d*_N_/*d*_S _ratios were estimated during the analysis with initial values of 2 and 0.4, respectively. Standard errors for total branch lengths (*t*) were reported by PAML, and these values were propagated to calculate standard errors for their corresponding *d*_S _and *d*_N _branch lengths according to the formulas

σdN=(N+S)σtN+Sω
 MathType@MTEF@5@5@+=feaafiart1ev1aaatCvAUfKttLearuWrP9MDH5MBPbIqV92AaeXatLxBI9gBaebbnrfifHhDYfgasaacH8akY=wiFfYdH8Gipec8Eeeu0xXdbba9frFj0=OqFfea0dXdd9vqai=hGuQ8kuc9pgc9s8qqaq=dirpe0xb9q8qiLsFr0=vr0=vr0dc8meaabaqaciGacaGaaeqabaqabeGadaaakeaacqaHdpWCdaWgaaWcbaGaemizaq2aaSbaaWqaaiabd6eaobqabaaaleqaaOGaeyypa0ZaaSaaaeaacqGGOaakcqWGobGtcqGHRaWkcqWGtbWucqGGPaqkcqaHdpWCdaWgaaWcbaGaemiDaqhabeaaaOqaaiabd6eaojabgUcaRmaalaaabaGaem4uamfabaGaeqyYdChaaaaaaaa@3FD0@

σdS=(N+S)σtNω+S
 MathType@MTEF@5@5@+=feaafiart1ev1aaatCvAUfKttLearuWrP9MDH5MBPbIqV92AaeXatLxBI9gBaebbnrfifHhDYfgasaacH8akY=wiFfYdH8Gipec8Eeeu0xXdbba9frFj0=OqFfea0dXdd9vqai=hGuQ8kuc9pgc9s8qqaq=dirpe0xb9q8qiLsFr0=vr0=vr0dc8meaabaqaciGacaGaaeqabaqabeGadaaakeaacqaHdpWCdaWgaaWcbaGaemizaq2aaSbaaWqaaiabdofatbqabaaaleqaaOGaeyypa0ZaaSaaaeaacqGGOaakcqWGobGtcqGHRaWkcqWGtbWucqGGPaqkcqaHdpWCdaWgaaWcbaGaemiDaqhabeaaaOqaaiabd6eaojabeM8a3jabgUcaRiabdofatbaaaaa@3FCA@

where *ω *is the *d*_N_/*d*_S _ratio and *N *and *S *are the number of nonsynonymous and synonymous sites, respectively, as reported by PAML. Branch lengths representing total substitutions per site for rRNA genes were estimated with baseml in PAML. The general time-reversible nucleotide model was used with a gamma distribution for rate variation with four categories. The rate matrix, nucleotide frequencies, and shape of the gamma parameter (initial value of 0.5) were estimated during the analysis.

All branch length analyses employed user-defined topologies. Within rosids, topologies were constrained according to the phylogenetic analysis carried out in this study (Fig. 3). Relationships in the Plantaginaceae were constrained according to [17]. All other relationships were constrained according to the Angiosperm Phylogeny Website [41].

### Absolute substitution rate estimates

Absolute rates of synonymous substitution per branch (*R*_S_) were calculated by dividing the synonymous branch length, *d*_S_, by the length of time, *T*, for that branch. Standard errors for *R*_S _were determined by propagating the errors associated with branch length and time according to the formula

σRS=RS(σdSdS)2+(σTT)2
 MathType@MTEF@5@5@+=feaafiart1ev1aaatCvAUfKttLearuWrP9MDH5MBPbIqV92AaeXatLxBI9gBaebbnrfifHhDYfgasaacH8akY=wiFfYdH8Gipec8Eeeu0xXdbba9frFj0=OqFfea0dXdd9vqai=hGuQ8kuc9pgc9s8qqaq=dirpe0xb9q8qiLsFr0=vr0=vr0dc8meaabaqaciGacaGaaeqabaqabeGadaaakeaacqaHdpWCdaWgaaWcbaGaemOuai1aaSbaaWqaaiabdofatbqabaaaleqaaOGaeyypa0JaemOuai1aaSbaaSqaaiabdofatbqabaGcdaGcaaqaamaabmaabaWaaSaaaeaacqaHdpWCdaWgaaWcbaGaemizaq2aaSbaaWqaaiabdofatbqabaaaleqaaaGcbaGaemizaq2aaSbaaSqaaiabdofatbqabaaaaaGccaGLOaGaayzkaaWaaWbaaSqabeaacqaIYaGmaaGccqGHRaWkdaqadaqaamaalaaabaGaeq4Wdm3aaSbaaSqaaiabdsfaubqabaaakeaacqWGubavaaaacaGLOaGaayzkaaWaaWbaaSqabeaacqaIYaGmaaaabeaaaaa@4701@

*R*_N _values and their standard errors were calculated similarly.

## Abbreviations

*d*_S _– number of substitutions per synonymous site; *d*_N _– number of substitutions per nonsynonymous site; *R*_S _– absolute rate of synonymous substitution per branch; *R*_N _– absolute rate of nonsynonymous substitutions per branch; ML – maximum likelihood; Myr – million years ago; NT – nucleotides; SSB – substitutions per site per billion years; SSU rDNA – small subunit ribosomal DNA; ti/tv – transition/transversion

## Authors' contributions

CLP generated much of the data newly reported in this study, conducted intial analyses, and helped with tables and manuscript revision in response to reviewers' comments. JPM generated some of the data, carried out all final analyses, prepared the figures and tables, and drafted part of the manuscript. YLQ, AJH, KS, and NDY contributed some of the data for this study. CWD guided some of the data generation and contributed to the manuscript preparation. JDP guided the entire study and drafted most of the manuscript. All authors read and approved the final manuscript.

## Supplementary Material

Additional File 1 **Absolute substitution rates per branch. **This table gives a complete list of estimates for divergence times, number of substitutions per synonymous site (*d*_S_), absolute rate of synonymous substitution (*R*_S_), number of substitutions per nonsynonymous site (*d*_N_), absolute rate of nonsynonymous substitution (*R*_N_), and *R*_N_/*R*_S _ratios for all branches shown in figure 4. Click here for file

Additional File 2 **Sequences used in this study. **This table lists the GenBank accession numbers for all sequences used in this study. 2a) List of mitochondrial sequences used for figure 1. 2b) List of chloroplast sequences used for figure 1. 2c) List of nuclear sequences used for figure 1. 2d) List of sequences used for figures 2, 3, 4. Click here for file

## References

[B1] Wolfe KH, Li WH, Sharp PM (1987). Rates of nucleotide substitution vary greatly among plant mitochondrial, chloroplast, and nuclear DNAs. Proc Natl Acad Sci USA.

[B2] Palmer JD, Herbon LA (1988). Plant mitochondrial DNA evolves rapidly in structure, but slowly in sequence. J Mol Evol.

[B3] Wolfe KH, Andersson B, Salter AH, Barber J (1996). Molecular evolution of plants: more genomes, fewer generalities. Molecular Genetics of Photosynthesis.

[B4] Gaut BS, Hecht MK, MacIntyre RJ, Clegg MT (1998). Molecular clocks and nucleotide substitution rates in higher plants. Evolutionary Biology.

[B5] Muse SV (2000). Examining rates and patterns of nucleotide substitution in plants. Plant Mol Biol.

[B6] Eyre-Walker A, Gaut BS (1997). Correlated rates of synonymous site evolution across plant genomes. Mol Biol Evol.

[B7] Laroche J, Li P, Maggia L, Bousquet J (1997). Molecular evolution of angiosperm mitochondrial introns and exons. Proc Natl Acad Sci USA.

[B8] Laroche J, Bousquet J (1999). Evolution of the mitochondrial *rps3 *intron in perennial and annual angiosperms and homology to *nad5 *intron 1. Mol Biol Evol.

[B9] Whittle CA, Johnston MO (2002). Male-driven evolution of mitochondrial and chloroplastidial DNA sequences in plants. Mol Biol Evol.

[B10] Barraclough TG, Savolainen V (2001). Evolutionary rates and species diversity in flowering plants. Evolution.

[B11] Jobson RW, Albert VA (2002). Molecular rates parallel diversification contrasts between carnivorous plant sister lineages. Cladistics.

[B12] Qiu YL, Cho Y, Cox JC, Palmer JD (1998). The gain of three mitochondrial introns identifies liverworts as the earliest land plants. Nature.

[B13] Cho Y, Qiu YL, Kuhlman P, Palmer JD (1998). Explosive invasion of plant mitochondria by a group I intron. Proc Natl Acad Sci USA.

[B14] Adams KL, Daley DO, Qiu YL, Whelan J, Palmer JD (2000). Repeated, recent and diverse transfers of a mitochondrial gene to the nucleus in flowering plants. Nature.

[B15] Palmer JD, Adams KL, Cho Y, Parkinson CL, Qiu YL, Song K (2000). Dynamic evolution of plant mitochondrial genomes: mobile genes and introns, and highly variable mutation rates. Proc Natl Acad Sci USA.

[B16] Adams KL, Qiu YL, Stoutemyer M, Palmer JD (2002). Punctuated evolution of mitochondrial gene content: high and variable rates of mitochondrial gene loss and transfer during angiosperm evolution. Proc Natl Acad Sci USA.

[B17] Cho Y, Mower JP, Qiu YL, Palmer JD (2004). Mitochondrial substitution rates are extraordinarily elevated and variable iHhin a genus of flowering plants. Proc Natl Acad Sci USA.

[B18] Bakker FT, Culham A, Hettiarachi P, Touloumenidou T, Gibby M (2004). Phylogeny of *Pelargonium *(Geraniaceae) based on DNA sequences of three genomes. Taxon.

[B19] Brown WM, Prager EM, Wang A, Wilson AC (1982). Mitochondrial DNA sequences of primates: tempo and mode of evolution. J Mol Evol.

[B20] Mower JP (2005). PREP-Mt: predictive RNA editor for plant mitochondrial genes. BMC Bioinformatics.

[B21] Malek O, Lattig K, Hiesel R, Brennicke A, Knoop V (1996). RNA editing in bryophytes and a molecular phylogeny of land plants. EMBO J.

[B22] Bowe LM, Coat G, dePamphilis CW (2000). Phylogeny of seed plants based on all three genomic compartments: extant gymnosperms are monophyletic and Gnetales' closest relatives are conifers. Proc Natl Acad Sci USA.

[B23] Chaw SM, Parkinson CL, Cheng Y, Vincent TM, Palmer JD (2000). Seed plant phylogeny inferred from all three plant genomes: monophyly of extant gymnosperms and origin of Gnetales from conifers. Proc Natl Acad Sci USA.

[B24] Davis JI, Stevenson DW, Peterson G, Seberg O, Campbell LM, Freudenstein JV, Goldman DH, Hardy CR, Michelangeli FA, Simmons MP, Specht CD, Vergara-Silva F, Gandolfo M (2004). A phylogeny of the monocots, as inferred from rbcL and atpA sequence variation, and a comparison of methods for calculating jackknife and bootstrap values. Syst Bot.

[B25] Wikstrom N, Pryer KM (2005). Incongruence between primary sequence data and the distribution of a mitochondrial *atp1 *group II intron among ferns and horsetails. Mol Phylogenet Evol.

[B26] Nickrent DL, Blarer A, Qiu YL, Vidal-Russell R, Anderson FE (2004). Phylogenetic inference in Rafflesiales: the influence of rate heterogeneity and horizontal gene transfer. BMC Evol Biol.

[B27] Beckert S, Steinhauser S, Muhle H, Knoop V (1999). A molecular phylogeny of bryophytes based on nucleotide sequences of the mitochondrial *nad5 *gene. Plant Syst Evol.

[B28] Foury F, Hu J, Vanderstraeten S (2004). Mitochondrial DNA mutators. Cell Mol Life Sci.

[B29] Miller JH (1996). Spontaneous mutators in bacteria: insights into pathways of mutagenesis and repair. Ann Rev Microbiol.

[B30] Horst JP, Wu TH, Marinus MG (1999). *Escherichia coli *mutator genes. Trends Microbiol.

[B31] Bousquet J, Strauss SH, Doerksen AH, Price RA (1992). Extensive variation in evolutionary rate of *rbcL *gene sequences among seed plants. Proc Natl Acad Sci USA.

[B32] Croteau DL, Bohr VA (1997). Repair of oxidative damage to nuclear and mitochondrial DNA in mammalian cells. J Biol Chem.

[B33] Yakes FM, Van Houten B (1997). Mitochondrial DNA damage is more extensive and persists longer than nuclear DNA damage in human cells following oxidative stress. Proc Natl Acad Sci USA.

[B34] Bowe LM, dePamphilis CW (1996). Effects of RNA editing and gene processing on phylogenetic reconstruction. Mol Biol Evol.

[B35] Adams KL, Song K, Roessler PG, Nugent JM, Doyle JL, Doyle JJ, Palmer JD (1999). Intracellular gene transfer in action: dual transcription and multiple silencings of nuclear and mitochondrial cox2 genes in legumes. Proc Natl Acad Sci USA.

[B36] Swofford DL (2002). PAUP*: phylogenetic analysis using parsimony (* and other methods).

[B37] Sanderson MJ (2003). r8s: inferring absolute rates of molecular evolution and divergence times in the absence of a molecular clock. Bioinformatics.

[B38] Schneider H, Schuettpelz E, Pryer KM, Cranfill R, Magallon S, Lupia R (2004). Ferns diversified in the shadow of angiosperms. Nature.

[B39] Leebens-Mack J, Raubeson LA, Cui L, Kuehl JV, Fourcade MH, Chumley TW, Boore JL, Jansen RK, dePamphilis CW (2005). Identifying the basal angiosperm node in chloroplast genome phylogenies: sampling one's way out of the Felsenstein Zone. Mol Biol Evol.

[B40] Yang Z (1997). PAML: a program package for phylogenetic analysis by maximum likelihood. Comput Appl BioSci.

[B41] Angiosperm Phylogeny Website. http://www.mobot.org/MOBOT/research/APweb/welcome.html.

