# Supplementary material for: Multiple major increases and decreases in mitochondrial substitution rates in the plant family Geraniaceae
Source: BMC Evol Biol. 2005 Dec 20;5:73. doi: 10.1186/1471-2148-5-73 (PMC1343592; doi:10.1186/1471-2148-5-73)
Supplement: Additional File 1 — Absolute substitution rates per branch. This table gives a complete list of estimates for divergence times, number of substitutions per synonymous site (dS), absolute rate of synonymous substitution (RS), number of substitutions per nonsynonymous site (dN), absolute rate of nonsynonymous substitution (RN), and RN/RS ratios for all branches shown in figure 4. [file 1471-2148-5-73-S1.pdf]

Additional file 1. *cox1* absolute substitution rates

Branch	Time (Myr)	d_S (sub./site x 100)	R_S (SSB)	d_N (sub./site x 100)	R_N (SSB)	R_N / R_S
A to B	6 ± 2	0.00 ± 0.01	0	0.00 ± 0.00	0	–
B to C	33 ± 4	0.44 ± 0.13	0.14 ± 0.04	0.98 ± 0.30	0.30 ± 0.10	2.23
C to Malp	72 ± 4	4.54 ± 1.15	0.63 ± 0.16	0.10 ± 0.02	0.01 ± 0.00	0.02
C to Popu	72 ± 4	4.01 ± 0.77	0.56 ± 0.11	1.39 ± 0.27	0.19 ± 0.04	0.35
B to D	7 ± 3	0.00 ± 0.00	0	0.20 ± 0.14	0.29 ± 0.23	–
D to E	3 ± 4	0.00 ± 0.01	0	0.00 ± 0.00	0	–
E to Betu	94 ± 3	1.62 ± 0.49	0.17 ± 0.05	0.49 ± 0.15	0.05 ± 0.02	0.30
E to Rham	94 ± 3	2.29 ± 0.69	0.24 ± 0.07	0.31 ± 0.09	0.03 ± 0.01	0.14
D to F	6 ± 4	0.00 ± 0.00	0	0.09 ± 0.10	0.14 ± 0.18	–
F to Cucu	91 ± 3	6.22 ± 1.15	0.69 ± 0.13	0.93 ± 0.17	0.10 ± 0.02	0.15
F to G	39 ± 4	2.39 ± 0.57	0.61 ± 0.16	0.91 ± 0.22	0.23 ± 0.06	0.38
G to Glyc	51 ± 3	1.35 ± 0.52	0.26 ± 0.10	0.19 ± 0.07	0.04 ± 0.01	0.14
G to Pisu	51 ± 3	1.64 ± 0.55	0.32 ± 0.11	0.29 ± 0.10	0.06 ± 0.02	0.18
A to H	4 ± 2	0.00 ± 0.01	0	0.00 ± 0.00	0	–
H to I	2 ± 3	0.00 ± 0.01	0	0.00 ± 0.00	0	–
I to Oeno	104 ± 2	3.59 ± 0.66	0.35 ± 0.06	1.67 ± 0.31	0.16 ± 0.03	0.47
I to J	9 ± 4	0.00 ± 0.00	0	0.09 ± 0.10	0.10 ± 0.12	–
J to Burs	95 ± 4	3.18 ± 0.93	0.34 ± 0.10	0.10 ± 0.03	0.01 ± 0.00	0.03
J to K	76 ± 4	5.56 ± 0.90	0.73 ± 0.12	1.89 ± 0.31	0.25 ± 0.04	0.34
K to Arab	19 ± 2	0.28 ± 0.29	0.15 ± 0.15	0.00 ± 0.00	0	0.00
K to Bras	19 ± 2	1.39 ± 0.58	0.74 ± 0.31	0.10 ± 0.04	0.05 ± 0.02	0.07
H to L	8 ± 4	0.23 ± 0.27	0.31 ± 0.40	0.00 ± 0.00	0	0.00
L to Cros	99 ± 3	1.95 ± 0.45	0.20 ± 0.05	1.18 ± 0.27	0.12 ± 0.03	0.61
L to M	36 ± 6	8.75 ± 1.29	2.43 ± 0.53	2.05 ± 0.30	0.57 ± 0.12	0.23
M to Hyps	62 ± 5	6.86 ± 1.28	1.10 ± 0.22	0.95 ± 0.18	0.15 ± 0.03	0.14
M to N	16 ± 6	6.74 ± 1.90	4.09 ± 1.87	0.35 ± 0.10	0.21 ± 0.10	0.05
N to O	11 ± 5	1.06 ± 1.32	0.96 ± 1.27	0.10 ± 0.12	0.09 ± 0.12	0.09
O to P	26 ± 4	1.09 ± 0.55	0.42 ± 0.22	0.00 ± 0.00	0	0.00
P to Mons	9 ± 2	0.81 ± 0.33	0.91 ± 0.46	0.29 ± 0.12	0.33 ± 0.16	0.36
P to Sarc	9 ± 2	0.54 ± 0.27	0.61 ± 0.35	0.19 ± 0.10	0.21 ± 0.13	0.35
O to Q	6 ± 4	0.00 ± 0.01	0	0.00 ± 0.00	0	–
Q to R	11 ± 4	0.00 ± 0.01	0	0.00 ± 0.00	0	–
R to Ggra	18 ± 3	1.08 ± 0.44	0.59 ± 0.26	0.19 ± 0.08	0.10 ± 0.05	0.18
R to Gmac	18 ± 3	0.81 ± 0.31	0.44 ± 0.18	0.39 ± 0.15	0.21 ± 0.09	0.48
Q to S	5 ± 4	0.81 ± 0.47	1.63 ± 1.59	0.00 ± 0.00	0	0.00
S to Etri	24 ± 3	0.27 ± 0.16	0.11 ± 0.07	0.19 ± 0.11	0.08 ± 0.05	0.70
S to T	18 ± 3	0.27 ± 0.27	0.15 ± 0.16	0.00 ± 0.00	0	0.00
T to Echr	7 ± 1	0.00 ± 0.00	0	0.10 ± 0.10	0.15 ± 0.15	–
T to Epel	7 ± 1	0.00 ± 0.02	0	0.00 ± 0.00	0	–
N to U	23 ± 5	68.89 ± 7.53	30.25 ± 7.54	1.57 ± 0.17	0.69 ± 0.17	0.02
U to V	10 ± 4	7.28 ± 3.47	7.17 ± 4.55	0.65 ± 0.31	0.64 ± 0.41	0.09
V to Pcap	13 ± 2	1.38 ± 0.54	1.06 ± 0.46	0.29 ± 0.11	0.22 ± 0.10	0.21
V to W	8 ± 3	0.78 ± 0.48	0.96 ± 0.69	0.10 ± 0.06	0.12 ± 0.09	0.13
W to Pcot	5 ± 2	0.00 ± 0.00	0	0.49 ± 0.22	0.98 ± 0.54	–
W to Pren	5 ± 2	1.86 ± 0.59	3.74 ± 1.69	0.29 ± 0.09	0.58 ± 0.27	0.16
U to X	8 ± 5	31.33 ± 5.10	37.68 ± 23.17	0.92 ± 0.15	1.11 ± 0.68	0.03
X to Pcan	15 ± 3	16.96 ± 2.65	11.38 ± 3.15	0.98 ± 0.15	0.66 ± 0.18	0.06
X to Y	5 ± 4	6.73 ± 2.11	13.41 ± 12.61	0.22 ± 0.07	0.44 ± 0.41	0.03
Y to Pque	10 ± 3	4.72 ± 1.25	4.77 ± 1.87	0.21 ± 0.06	0.21 ± 0.08	0.04
Y to Z	8 ± 3	5.70 ± 1.39	7.25 ± 3.39	0.00 ± 0.00	0	0.00
Z to Palc	2 ± 1	0.24 ± 0.11	1.18 ± 0.95	0.38 ± 0.18	1.88 ± 1.49	1.58
Z to Phor	2 ± 1	1.03 ± 0.39	5.08 ± 3.78	0.33 ± 0.13	1.63 ± 1.21	0.32
