# Supplementary material for: Multiple major increases and decreases in mitochondrial substitution rates in the plant family Geraniaceae
Source: BMC Evol Biol. 2005 Dec 20;5:73. doi: 10.1186/1471-2148-5-73 (PMC1343592; doi:10.1186/1471-2148-5-73)
Supplement: Additional File 2 — Sequences used in this study. This table lists the GenBank accession numbers for all sequences used in this study. 2a) List of mitochondrial sequences used for figure 1. 2b) List of chloroplast sequences used for figure 1. 2c) List of nuclear sequences used for figure 1. 2d) List of sequences used for figures 2, 3, 4. [file 1471-2148-5-73-S2.pdf]

Additional file 2a. List of mitochondrial sequences used for figure 1

	GenBank Accession Numbers					
**Genus**	***atp1***	***cob***	***cox2***	***cox3***	**mt LSU**	***nad1***
Mahonia	–	AY832081	–	AY832113	–	–
Ranunculus	AF197714	–	–	–	–	AY674761
Platanus	AY009423	AY832084	–	AY832117	–	AY832123
Beta	BA000009	BA000009	BA000009	BA000009	BA000009	BA000009
Daucus	–	–	X63625	–	–	–
Sambucus	AY741813	DQ317064	–	DQ317066	–	–
Nicotiana	BA000042	BA000042	BA000042	BA000042	BA000042	BA000042
Chaerophyllum	–	–	–	–	–	AY283607
Pl. media	AY818938	–	–	–	–	–
Pl. rugelii	AY818939	AY818946	AY818947	–	AY818948	–
Oenothera	X04023	X07126	X00212	X04764	X02559	M63033
Glycine	Z14031	–	X04825	X15131	–	AJ428875
Lupinus	–	–	–	–	–	–
Pisum	D14698	AJ132231	AJ414385	–	–	–
Vicia	–	X07237	–	X51690	–	–
Arabidopsis	Y08502	Y08501	Y08501	Y08502	Y08501	Y08502
Brassica	AP006444	AP006444	AP006444	AP006444	AP006444	AP006444
Hypseocharis	DQ317060	–	–	–	–	–
Geranium	–	–	–	–	–	AF167132
Sarcocaulon	DQ317062	–	–	–	–	AF167133
E. pelargoniflorum	DQ317061	–	–	–	–	–
Pe. hortorum	DQ317063	DQ317065	DQ317069	DQ317067	DQ317059	–
Pe. tongaense	–	–	–	–	–	AF167104
Pe. cotyledonis	–	–	–	–	–	AF256578
Pe. reniforme	–	–	–	DQ317068	–	–

GenBank accession numbers for *cox1* and mt SSU are listed in additional file 2d.

Additional file 2b. List of chloroplast sequences used for figure 1

	GenBank Accession Numbers			
**Genus**	***matK***	***ndhF***	***petD***	***rps11***
Clematis	–	AY145147	–	–
Ranunculus	DQ069586	–	DQ069568	DQ069538
Platanus	AF543747	AY394743	–	–
Spinacia	AJ400848	AJ400848	AJ400848	AJ400848
Panax	AY582139	AY582139	AY582139	AY582139
Nicotiana	Z00044	Z00044	Z00044	Z00044
Pl. rugelii	DQ317070	AY818915	–	–
Oenothera	AJ271079	AJ271079	AJ271079	AJ271079
Lotus	AP002983	AP002983	AP002983	AP002983
Pisum	AY386961	–	AF153442	–
Vicia	–	X14804	–	–
Vigna	–	–	–	AF536226
Arabidopsis	AP000423	AP000423	AP000423	AP000423
Brassica	AY541618	AF064647	–	–
Pe. hortorum	DQ317071	DQ317072	DQ317073	DQ317074

GenBank accession numbers for *rbcL* are listed in additional file 2d.

Additional file 2c. List of nuclear sequences used for figure 1

Genus	GenBank Accession Numbers		
	aco	***acs***	***etr***
Delphinium	–	–	AB201245
Dianthus	L35152	–	AB035806
Rumex	Y10034	AF038945	–
Helianthus1	U62553	–	–
Helianthus2	U62554	–	–
Lactuca1	–	AF380836	AF350321
Lactuca2	–	AF380837	–
Nicotiana1	AB012857	AJ005002	AF022727
Nicotiana2	X83229	X65982	–
Antirrhinum	–	AF083814	AY159362
Pl. major	AJ843131	–	–
Striga	–	AB072042	–
Glycine	–	X67100	–
Lupinus	–	AF119411	–
Pisum	–	AB049725	–
Trifolium1	AF115262	–	–
Trifolium2	AF115261	–	–
Vigna1	AB002667	Z11613	–
Vigna2	AF315316	–	–
Fagus	–	–	AJ420193
Prunus	–	–	AF124527
Mangifera1	AJ297435	AF170705	AF227742
Mangifera2	AJ505610	U22523	–
Arabidopsis1	NM_100380	NM_116016	NM_105305
Arabidopsis2	NM_101073	–	–
Brassica1	AF252853	X82273	AF047476
Brassica2	X81629	–	–
Carica1	–	U68216	–
Carica2	–	AF178077	–
Pe. hortorum1	U07953	U88971	AF141928
Pe. hortorum2	U19856	U17229	–

Additional file 2d. List of sequences used for figures 2-4

	GenBank Accession Numbers					
**Genus**	***cox1***	**mt SSU**	***atpB***	***matK***	***rbcL***	**nc SSU**
Euptelea	AF193963	AF193997	–	–	AY048174	L75831
Ranunculus	DQ317030	AF161093	–	–	L08766	L24092
Grevillea	AF193961	AF193995	–	–	AF197589	AF197577
Platanus	DQ317031	AF161090	–	–	unpublished	U42794
Tetracentron	AF193964	AF193998	–	–	L12668	U42814
Trochodendron	AF020581	AF161092	–	–	L01958	U42816
Buxus	AF193962	AF193996	–	–	AF093717	L54065
Beta	BA000009	AF161094	DQ067451	AY514832	AY270065	AF161096
Plumbago	DQ317032	–	–	–	M77701	U42795
Spinacia	DQ317033	–	–	–	V00168	L24420
Diospyros	AJ223417	–	AF213768	AY145446	L12613	L49279
Symplocos	AJ223435	–	–	–	L12624	U43297
Ilex	AJ223429	–	–	–	AY725859	AF206938
Sambucus	AF193965	AF194000	–	–	AJ420867	AF193941
Nerium	AJ223421	–	–	–	AJ002886	AF107572
Nicotiana	unpublished	AF161095	X61319	Z00044	unpublished	AJ236016
Solanum	X83206	AJ252732	–	–	M76402	X67238
Jasminum	AJ247607	–	–	–	L01929	AJ236035
Lamium	AJ223428	–	–	–	Z37399	L49287
Sesamum	AJ247598	–	AJ236176	AJ429340	L14408	AJ236041
Epifagus	–	U82642	–	–	–	–
Digitalis	AJ223415	AF193999	–	–	X83720	AF193940
Veronica	AJ223427	AY818950	–	–	AJ247611	AF207052
Pl. atrata	AJ389610	AJ389618	–	–	AJ389601	–
Pl. lanceolata	AJ389611	AJ389619	–	–	L36454	AJ236046
Pl. sempervirens	AJ389612	AJ389620	–	–	AJ389602	–
Pl. sericea	AJ389613	AJ389621	–	–	AJ389603	–
Pl. coronopus	AJ389609	AJ389617	–	–	AJ389600	–
Pl. media	AJ389605	AJ389614	–	–	AJ389596	–
Pl. australis	AJ389608	AY818949	–	–	AJ389599	–
Pl. rigida	AJ389607	AJ389616	–	–	AJ389598	–
Pl. rugelii	AJ389606	AJ389615	–	–	AJ389597	AY818951
Oenothera	X05465	X61277	AJ271079	AJ271079	Werman et al.	U67930
Malpighia	AJ223433	–	AJ235527	AF344561	AJ247619	L24046
Populus	U77623	–	AF209658	AJ506088	AF206812	AF206999
Maytenus	–	AY855839	–	–	–	–
Cucumis	AJ223416	AY258278	AJ970307	AJ970307	AF206755	AF206894
Glycine	M16884	M16859	–	AF142700	Z95552	X02623
Pisum	X14409	–	X03852	AY386961	X03853	U43011
Lupinus	–	Z11512	–	–	–	–
Betula	U77620	–	AJ235411	AY372022	X56617	L00971
Rhamnus	AJ223422	–	AJ235579	AY257533	L13189	AJ235979
Arabidopsis	Y08501	Y08501	AP000423	AP000423	unpublished	X16077
Brassica	AP006444	AP006444	AF267641	AY54161	M88342	–
Raphanus	–	–	–	–	–	unpublished
Bursera	AJ223412	–	AF035899	–	L01890	AF206877
Cytinus	–	U82639	–	–	–	–

Crossosoma	DQ317034	AF194001	AF209571	–	L11179	AF193942
Hypseocharis	AF193967	AF194002	–	–	DQ317048	DQ317014
G. grandiflorum	AF193968	AF194003	–	–	L01920	AF193943
G. macrorhizum	DQ317035	DQ317028	–	–	DQ317049	DQ317015
Monsonia	DQ317036	DQ317029	AF209632	–	L14701	DQ317016
Sarcocaulon	DQ317037	DQ317004	–	–	L14705	DQ317017
E. chrysanthum	DQ317038	DQ317005	–	–	DQ317050	DQ317018
E. pelargoniflorum	DQ317039	DQ317006	–	–	DQ317051	DQ317019
E. trifolium	DQ317040	DQ317007	–	–	DQ317052	DQ317020
Pe. cotyledonis	DQ317041	DQ317008	AF035911	–	DQ317053	DQ317021
Pe. reniforme	DQ317042	DQ317009	–	–	DQ317054	DQ317022
Pe. capitatum	DQ317043	DQ317010	–	–	L14702	DQ317023
Pe. candicans	DQ317044	DQ317011	–	–	DQ317055	DQ317024
Pe. quercetorum	DQ317045	–	–	–	DQ317056	DQ317025
Pe. alchemilloides	DQ317046	DQ317012	–	–	DQ317057	DQ317026
Pe. hortorum	DQ317047	DQ317013	–	DQ317071	DQ317058	DQ317027
